# *Helicobacter pylori*-Mediated Oxidative Stress and Gastric Diseases: A Review

**DOI:** 10.3389/fmicb.2022.811258

**Published:** 2022-02-08

**Authors:** Lu Han, Xu Shu, Jian Wang

**Affiliations:** ^1^Department of Gastroenterology, The First Affiliated Hospital of Nanchang University, Nanchang, China; ^2^Jiangxi Clinical Research Center for Gastroenterology, The First Affiliated Hospital of Nanchang University, Nanchang, China

**Keywords:** *Helicobacter pylori*, gastric diseases, virulence factors, oxidative stress, promising-therapy

## Abstract

Gastric cancer is considered to be a type of gastrointestinal tumor and is mostly accompanied by *Helicobacter pylori* (HP) infection at the early stage. Hence, the long-term colonization of the gastric mucosa by HP as a causative factor for gastrointestinal diseases cannot be ignored. The virulence factors secreted by the bacterium activate the signaling pathway of oxidative stress and mediate chronic inflammatory response in the host cells. The virulence factors also thwart the antibacterial effect of neutrophils. Subsequently, DNA methylation is induced, which causes continuous cell proliferation and evolution toward low-grade-differentiated gastric cells. This process provides the pathological basis for the occurrence of progressive gastric cancer. Therefore, this review aims to summarize the oxidative stress response triggered by HP in the gastric mucosa and the subsequent signaling pathways. The findings are expected to help in the formulation of new targeted drugs for preventing the occurrence of early gastric cancer and its progression to middle and advanced cancer.

## Introduction

The prevalence of infection by *Helicobacter pylori* (HP), a Gram-negative microaerobic bacterium, differs significantly between developed and developing countries (Fock and Ang, 2010). Recently, the frequency of HP infection and the related diseases has demonstrated a decreasing trend in the Western countries, but it has shown an increasing trend every year with age in China, especially after the age of 10 years, which is closely related to the socioeconomic status and antibiotic resistance in China (Malfertheiner et al., 2017). Statistical data has demonstrated that HP infection occurs during childhood and develops into a hidden long-term infection or, later, into a variety of diseases. These diseases can be classified into internal and external gastrointestinal diseases. The former is mainly believed to be chronic gastritis, which is the most common one (Blaser, 1999). The initial gastritis can proceed to chronic non-atrophic, active, or atrophic gastritis. Approximately 10%–15% of HP-infected patients suffer from sinus gastritis, which may also be associated with their own concomitant hypergastrinemia (Censini et al., 2001). Eventually, the patients are prone to develop duodenal ulcers, even intestinal metaplasia with dysplasia, and non-cardia intestinal-type gastric adenocarcinoma and sporadic diffuse gastric cancer (Compare et al., 2010). When HP adheres to the underlying epithelium, it can induce gastric mucosa-associated lymphoid tissue (MALT) lymphoma or gastric lymphoma adenocarcinoma (Venerito et al., 2016; Kalisperati et al., 2017; Zhang et al., 2017). Interestingly, growing evidence suggests that HP is also strongly associated with extra-gastric diseases (Zhu et al., 2016), including neurological, cutaneous, hematological, ocular, cardiovascular, metabolic, allergic, and hepatobiliary diseases. However, gastritis and peptic ulcers are predominant gastric diseases. To prevent the development of gastric diseases triggered by HP infection at an early stage, it is critical to understand the pathogenic mechanism of HP in the gastric host cells (GHCs). Generally, the effect of self-immune response can eliminate some bacteria and block their propagation in the gastric mucosal layer. However, HP possesses a self-protection mechanism, which not only neutralizes the damage caused by gastric acid through ammonia but also simulates the “proton channel” by Urel protein to suppress the release of gastric acid during colonization. Interestingly, the virulence factors of HP play a significant role in destroying the GHCs (Parker et al., 2010), such as cytotoxin-associated gene A (CagA), vacuolized cytotoxin (VacA; Backert and Clyne, 2011), the blood group antigen binding adhesion (BabA), etc. Presently, eradicating HP with the increase of antibiotic resistance has become a challenging solution. In this study, we investigated the oxidative stress-mediated signaling pathway by virulence factor of HP, which provides theoretical guidance for the eradication of HP in combination with novel-targeted and other alternative therapies.

## *Helicobacter pylori* Virulence Factors

The virulence factors of HP are attributed to three pathological mechanisms that maintain the infected status in the GHCs, including colonization (participated in urease, adhesin, and flagellum chemotactic system), immune escape [i.e., lipopolysaccharides (LPS), flagellum, CagA, and VacA], and disease induction (VacA, BabA, and DupA; Sicinschi et al., 2012). The abovementioned pathological processes contribute to the generation of a chronic inflammatory environment at the GHCs and facilitate HP colonization, thus providing the pathological basis for the development of early gastric cancer, namely, CagA, VacA, and outer membrane proteins (OMPs) that include HomB, HopQ, HopH (OipA), HopZ, HopP (SabA), and HopS (BabA; Espinoza et al., 2018). The regulation of specific molecular mechanisms has been described in this section. The regulatory mechanisms of the aforementioned virulence factors and their downstream-related signaling pathways associated with oxidative stress are described in this section.

The physiological processes of CagA mediated by HP infection are closely associated with apoptosis, HP movement elongation, cell adhesion, and oxidative stress (González et al., 2011). These findings revealed the higher expression level of regeneration gene 3 (reg3) in gastric cancer cells with HP-CagA^+^. In addition, CagA and reg3 both contribute synergistically to the G1/S phase transition of the cell cycle through the CDK4/CyclinD1 complex (Liu et al., 2019). Simultaneously, CagA is able to translocate into the intracytoplasmic space of the GHCs with the help of type-IV secretion system (T4SS). After the entry of CagA into the cytosol, it phosphorylates complexine, and both of which forms a trimer with SHP-2 molecules, modulating the oncogenic effect of HP infection. Moreover, CagA regulates the transcriptional levels in a non-phosphorylated manner, interacting with PLC-γ to stimulate calcium-dependent serine/threonine calmodulin phosphatase, and to induce the activation of nuclear factor of activated T cells (NFAT), which allows the translocation of the nucleus to resist the glutathione (GSH) defense systems (Nam et al., 2004). The abovementioned processes cause the accumulation of hydrogen peroxide and reactive oxygen species (ROS) in the organisms, which in turn causes DNA base damage with the production of single-stranded breaks and subsequently increases chromosomal instability, thereby providing a pathological basis for the development gastric carcinogenesis (den Hartog et al., 2013; Hanada et al., 2014; Liu et al., 2019). CagA directly induces a severe inflammatory response in the stomach with elevated levels of IL-37 (Yucel, 2019; Davarpanah et al., 2020). Furthermore, it promotes the invasiveness and migration of gastric adenocarcinoma cells (AGS) by activating the NOD-like receptor (Nod) thermal protein domain-associated protein 3 (NLRP3) inflammasome pathway and upregulating zincfinger ebox binding homeobox 1 (ZEB1) transcription, claudin-2, and the caudal-type homeobox transcription factor (CDX-2) expression (Jin et al., 2020). Strikingly, the specific pathogenic island of cytotoxin-associated gene (Cag) is called CagPAI, which encodes a CagA protein of 120–140 kDa that is considered to be a factor assisting in immune evasion. The increased expression of CagPAI appears to cause gastric cancer (Chen et al., 2016). The above-mentioned evidence suggests that HP strains carrying CagA-positive genes can significantly increase the risk of gastric mucosal carcinogenesis.

Vacuolized cytotoxin is considered to be another virulence factor that induces mitochondria damage mediated by the signaling pathway of β-linked protein, following the intracellular Ca^2+^ influx and a large accumulation of ROS. Moreover, this process would activate nuclear factor NF-kB to set the stage for promoting oxidative stress (Kim et al., 2007). VacA has some special regions mediating gene expression, including –S1 (–S1a, –S1b, and –S1c), –S2, –I1, –I2, –M1, and –M2 isoforms (Wang et al., 2014), and is capable of directly affecting the activity of vacuoles between HP strains. For example, the submits –S1, –I1, and –M1 of VacA are regarded to have a stronger ability to liquefy vacuoles than the –S2, –I2, and –M2 subunits (Touati et al., 2003), which contribute to the chemotaxis of gastric mucosal epithelial cells toward the intestinal epithelium in rat models (Sheh et al., 2010). In addition, it affects the cell migration and differentiation of the GHCs (Bussière et al., 2005). Recent studies have indicated that VacA, a key toxin of inducing autophagy and apoptosis, can be considered as a marker to predict the risk of gastric carcinogenesis in animal models (Carlosama-Rosero et al., 2021; Luo et al., 2021).

**Figure 1:**
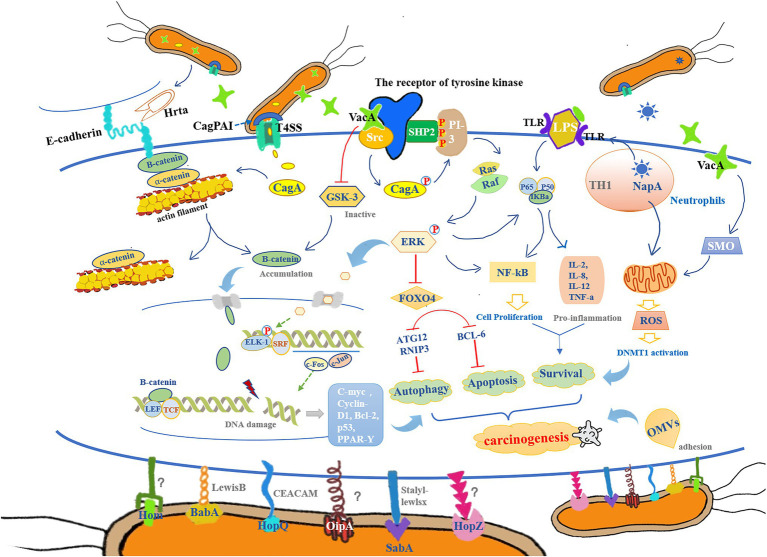
The colonization and reproduction of *Helicobacter pylori* (HP) are closely related to its chemotaxis, adhesion, colonization, virulence, inflammation, and host immune tolerance. It mediates the inflammatory response in the gastric epithelium through pathological processes such as oxidative stress, DNA methylation, and cell proliferation, which subsequently cause DNA damage, autophagy, apoptosis, and abnormal cell proliferation, to ultimately provide a pathological basis for cancer development. (1) Cytotoxin-associated gene (cag) pathogenicity island (CagPAI) can contribute to the opening of the type-IV secretion system (T4SS) channels and assist in the release of cytotoxin-associated gene A (CagA) from HP into gastric mucosal epithelial cells, thereby promoting the breakdown of β-catenin/a-catenin and actin filament complexes, leading to β-catenin accumulation. In addition, vacuolized cytotoxin (VacA) enters the gastric epithelial cells and further promotes β-catenin accumulation by inactivating GSK-3. When β-catenin enters into the nucleus, it forms a complex with LEF and TCF, thereby activating the expression of genes encoding cyclin D1 (cyclin-D1) and c-Myc, which leads to abnormal cell proliferation. In addition, CagA mediates the NF-kB/ERK/FOXO4 pathway to regulate the apoptotic and autophagic processes in gastric epithelial cells, which is in concert with other pro-inflammatory responses involved in the regulation of physiological processes of cell proliferation that leads to the malignant transformation of gastric mucosal epithelial cells to cancer cells. (2) Hrta secreted by HP, can cut E-calmodulin, disrupt intercellular adhesion, and lose cell polarity, providing conditions for HP colonization. Meanwhile, BabA, SabA, and HopQ can bind to their corresponding receptors on the mucus layer (LewisB, Stalyl–Lewlsx, and CEACAM, respectively), while the receptors of OipA, HopZ, and Hom remain uncleared. These outer membrane proteins (OMPs) promote the HP adhesion to host cells, and in combination with lipopolysaccharides (LPS)-binding complex, lead to the activation of NF-kB and other chemokines [IL-2 and interleukin-8 (IL-8)]. (3) VacA released by HP, can also bind to the receptors of tyrosine kinase, and cause Src to phosphorylate CagA, which triggers SHP2 to bind to PI-3 kinase, allowing the activation of Ras, Raf, and ERK. Phosphorylated ERK translocates to the nucleus with the activation of ELK1 and SRF complexes and induces c-Fos and c-Jun gene expressions. In addition, VacA also increases the activity of spermidine oxidase (SMO); both of these pathways can lead to DNA damage with a high expression of inflammatory factors. (4) NapA, another virulence factor of HP, is recruited by neutrophils that exert a significant role in releasing exogenous reactive oxygen species (ROS) in an attempt to clear the infection of HP, which in turn causes more damage in the gastric mucosa.

Outer membrane proteins, as a regulatory factor, not only mediate the interaction between bacteria and gastric mucosal epithelial cells but also perform the function of synergistically stimulating the two aforementioned virulence factors, CagA and VacA, to enhance the ability of immune escape (Figure 1). Moreover, different categories of OMPs possess their own physiological functions. For example, both BabA and HopQ facilitate the easy translocation of CagA into the GHCs by adhering to the gastric mucosal epithelial cells with the help of T4SS. They also trigger the inflammatory response by increasing the level of interleukin-8 (IL-8; Shahi et al., 2015; Moonens et al., 2018). The incidence of early gastric cancer was found to be significantly reduced in Cag-T4SS-deficient animal models (Sit et al., 2020). Another outer membrane protein, OipA, plays the main function of mediating the downstream signaling pathways through the phosphorylation of transcriptional activator-1 (STAT-1; Alarcón-Millán et al., 2019). It has also been reported that activated OipA increases the risk of gastric cancer and ulcer development, while its functional state depends intimately on the –s1/–m1 isoforms of CagA and VacA (Farzi et al., 2018). When the CagPAI sequence is intact, it promotes binding to OipA, which allows HP to be tightly positioned on the gastric mucosa with the development of inflammation. Although, HopZ is the only identified mucosal agent, HomB is also involved in the secretion of the proinflammatory factor IL-8 (Oleastro et al., 2008). IL-8 and tumor necrosis factor-α (TNF-α) can be used as markers of inflammation and oxidative stress (O'Hara et al., 2009). Notably, the seropositivity of OMPs directly affects the incidence of precancerous lesions and gastrocarcinoma, reaching up to four times more than that of the normal population (Cai et al., 2016; Epplein et al., 2018). Therefore, OMPs, in concert with virulence factors, synergistically support long-term HP infection and facilitate the oxidative stress response in GHCs (Cover, 2016; Braga et al., 2019), which lays the pathological foundation for the occurrence and development of gastric carcinogenesis.

DupA has been identified as a virulence factor that increases the risk of duodenal ulcer (Ansari and Yamaoka, 2017). The prevalence of DupA has been found to be higher in patients with early gastric cancer than in those with gastritis or gastric ulcer (Šterbenc et al., 2019). The available data on populations with a high incidence of ulcer carcinogenesis, such as Korea and Japan, were collected, which showed DupA-positivity rate as high as 48 and 21%, respectively (Miftahussurur et al., 2021). In addition, we found that it was less likely to detect DupA in patients with gastric ulcer without cancerous lesions (Miftahussurur et al., 2015). However, it is interesting to note that patients with duodenal ulcer who were DupA-positive were not as susceptible to gastric cancer as others (Yonezawa et al., 2013), mainly because of the presence of the short type of DupA (Alba et al., 2017). Moreover, the shorter gene of DupA exerted some protective effect on the gastric mucosa and decreased the rate of gastric mucosal carcinogenesis in the later stages (Takahashi et al., 2013). In fact, it has been reported that intact DupA (DupA1) is strongly associated with duodenal ulcer in India, instead of the short type of DupA1 cluster (Kim et al., 2018). Therefore, whether different subtypes of DupA can protect the patients with duodenal ulcer from early gastric cancer warrants further exploration.

Outer membrane vesicles (OMVs) are mainly enriched in non-featured proteins, flagellar basal body proteins, or OMPs (Olofsson et al., 2010). It has been reported to exert surface adhesion and chemotactic effects, which prevents HP from being destroyed by gastric acid and aids in the release of various inflammatory factors with the contribution of virulence factors (Jarzab et al., 2020). Of course, different subtypes of HP strain can directly affect the heterogeneity of OMVs proteins, subsequently regulating the adsorption, motility, acid tolerance, immune evasion, and protein intersection functions. Furthermore, OMVs can directly affect the activity of the body’s T-cells. An OMV inhibitor can effectively block the promotion of IL-10 and COX-2 released by HP in the monocytes. This observation suggests that OMV release provides a pathological basis for HP infection to achieve immune escape (Hock et al., 2017). Therefore, OMVs can also be considered as one of the risk factors for gastric precancerous lesions after HP infection.

Furthermore, a specific cholesterol regulatory enzyme exists in HP. The cholesterol-a-glucosyltransferase (CGT) encoding type-1 podocyte polysaccharide biosynthesis protein J (CapJ) induces cell membrane remodeling by tracking specific proteins on the cell membrane, ultimately causing autophagy in concert with CagA and VacA. When CGT is inhibited in the bacteria, the indirect inhibition of CapJ significantly reduces the production of cholesteryl glucoside. Meanwhile, it inhibits the opening of the T4SS channels, which in turn reduces the entry of CagA into the cell to inhibit the activation of the proinflammatory response (Wang et al., 2012).

In addition to being related to the abovementioned virulence factors, the infiltration and long-term infection of HP are both closely associated with urease, HP flagellum, heat shock protein (e.g., HSP, HSP10, and HSP60) as well as genetic polymorphisms of the host cells (Chen et al., 2016). Interestingly, the mutation of ATP4Ap.R703C leads to gastric acid deficiency, and the disturbance of pH balance can directly affect the physiological function of the mitochondrial respiratory chain, causing ROS signaling to be activated and triggering caspase-3-mediated apoptosis in the host cells (Benítez et al., 2020). Furthermore, HP infection elevates the pH of the gastric mucosa, which contributes to the proinflammatory response caused by R703C mutation and further exacerbates the oxidative stress process. If gastric acid is added externally to restore the pH balance, the mitochondrial function is largely re-established (Benítez et al., 2020). Indeed, the incidence of gastric cancer in the HP carriers varies across region (Choi et al., 2015). For example, the rate of HP infection is not proportional to the incidence of gastric cancer in Africa (da Costa et al., 2015; Chang et al., 2016). The researchers should further investigate the pathological mechanisms through which different virulence factor subtypes of HP promote gastric mucosal intestinal chemotaxis to understand the relationship between HP infection and the morbidity of gastric carcinoma.

## Oxidative Stress Generation

The levels of NO and reduced glutathione were both significantly lower in patients with HP than in the uninfected patients (controls). On the other hand, the levels of malondialdehyde, catalase, and superoxide dismutase were higher (Morishita et al., 2012). The specific mechanism of oxidative stress has been reported as follows: nicotinamide adenine dinucleotide phosphate (NADPH) oxidase (NOX) on the cell membrane, activated by upstream stimuli, provides electrons to oxygen and form superoxide (O_2_^−^). Furthermore, O_2_^−^ evolves into hydrogen peroxide (H_2_O_2_) catalyzed by superoxide dismutase and, in turn, forms the more toxic hypochlorous acid. Meanwhile, H_2_O_2_ can also react with O_2_^−^ to generate excess hydroxyl radicals (OH^−^). Accordingly, the abovementioned harmful substances are collectively referred to as ROS, which can destroy the majority of bacteria in the neutrophils and serve as a protective mechanism for the organism in the early stages (Shirin et al., 2001; Lu, 2013). However, the neutrophils of the tissue are unable to destroy the assembled colony of HP *via* paracrine secretion, thereby providing a condition for the long-term residence of HP. Moreover, it further induces chronic active inflammation of the gastric mucosa. It has been reported that a key enzyme, inducible nitric oxide synthase (iNOS), exists in the host neutrophils and epithelial cells, which catalyzes the formation of NO and later evolves with O_2_^−^ to peroxynitrite. This process allows further increase in ROS and reactive nitrogen species (RNS) formation and aggravates the oxidative stress response (Tsugawa et al., 2012). This mechanism has been confirmed in an animal model. For example, iNOS gene-deficient mice demonstrated a significant decrease in the incidence of gastric cancer after HP infection when compared with that in the wild-type mice. Accordingly, it has been hypothesized that oxidative stress induced by iNOS is inextricably associated with the developmental pathology of gastric cancer (Gmünder and Dröge, 1991). The process of oxidative stress is the consequence of the accumulation of ROS and RNS caused by neutrophils (Kang et al., 2004). Also, the levels of NO, reduced glutathione, catalase, and malondialdehyde can all respond to the oxidative stress in the gastric mucosa of patients with HP.

Currently, the critical role of killing HP is mainly played by neutrophils (phagocytes?), while epithelial cells have a weaker sterilizing effect on HP. The function of epithelial cells is to facilitate the redox signaling between host cells, which contributes to the development of early gastric cancer. Notably, polyamines produced from LPS in HP can promote apoptosis by phagosomes and inhibit the antioxidative process. In addition, superoxide radicals produced by NapA can inactivate the neutrophils and allow the microenvironment to evolve toward a chronic inflammatory process that favors long-term survival of HP and the development of early gastric cancer (Benaissa et al., 1996).

Presently, most studies are devoted to the examination of DNA methylation and tissue damage caused by oxidative stress (Pignatelli et al., 2001), although, the specific molecular mechanisms remain unclear. The involved signaling pathways are quite diverse, promoting not only the production of inflammatory factors (IL-8, IL-6, and IL-1β), but also the activation of granulocyte–macrophage colony-stimulating factor, monocyte chemotaxis protein-1 (CCL2), macrophage movement inhibitory factor (MIF), TNF-α, and tumor growth factor-β (TGF-β; Beswick et al., 2006). It has also been indicated that PRDX-2 can effectively scavenge the strongest antioxidant protein of ROS and H_2_O_2_ and protect the gastric mucosa from atypical proliferation under HP stimulation (Lei et al., 2016; Nicolussi et al., 2017). Therefore, this section intends to summarize the relevant signaling pathways involved in the oxidative stress caused by HP in recent years. Perhaps, targeted-therapy can provide a pathological basis for reducing the incidence of early gastric cancer (Esedov et al., 1999).

### Relative Pathway of Oxidative Stress Related to HP

First, CagA activates T-cell colonization in the GHCs through the epithelial-mesenchymal transition (EMT), which subsequently promotes the release of inflammatory factors such as TNF-β, NADH oxidase (NOX1), iNOS, IL-1, IL-8, and IL-10 (Zhang et al., 1998). High pH levels lay the foundation for the release of inflammatory factors. Recently, it has been reported that HP promotes neutrophil chemotaxis mainly due to the binding of the NAP binding sites in HP to TLR-2 on the epithelial cell membranes and the subsequent activation of the T-helper type 1 (Th1) immune system through the IL-17a/JAK2/STAT3 signaling pathway (Meliț et al., 2019). The abovementioned process activates the Nod1/NF-kB/MAPK/ERK/FOXO4 signaling pathway, thereby disrupting the programmed apoptosis of the cells, which is regarded as a protective mechanism in the organisms. Continuous progression toward intestinal epithelial chemotaxis ensues (He et al., 2020), which provides the pathological basis for the development of early gastric cancer. In parallel, the expression of oncogenes, such as p53, CDH1/E-cadherin, and adenomatous polyposis coli (APC), is significantly downregulated (Meliț et al., 2019). This process is accompanied by the inhibition of the tyrosine kinase signaling pathway and the involved genes, including those encoding receptor-associated tyrosine kinases and non-receptor kinases (Chichirau et al., 2019).

The above process is regulated by the CagA of HP and participate in the pathological regulatory process of early gastric cancer (Shi et al., 2014; Takahashi-Kanemitsu et al., 2020). When unphosphorylated CagA binds to e-linked protein (CDH-1), it predisposes the latter to nuclear translocation in gastric epithelial cells and subsequently activates the β-linked protein-dependent oncogenic pathway [e.g., cyclind1, c-MYC, and caudal-type homeobox1 (CDX1)], promoting gastric mucosa to intestinal chemotaxis. Posteriorly, the release of lysogenic connexins activates the EGFR/ErbB receptors and mediates the activation of the Ras/MEK/Erk signaling pathway (Devaux et al., 2019). It has also been suggested that CagA is associated with the hypermethylation of adhesion proteins, such as CDH-1. If HP is eliminated, not only is the DNA methylation level of the organism comparable to the normal level but the oxidative stress response is also diminished. Early studies have stated that DNA methylation is common in diseases such as hematological malignancies (AML) and digestive tract cancers (Chan et al., 2006; Cimmino et al., 2018; Pawlowska et al., 2019). The process mainly involves chronic inflammation mediated by the activation of TGF-B and NF-kB signaling. Moreover, NF-kB has the property of activating downstream exogenous and endogenous carcinogens (Taniguchi and Karin, 2018), which promotes the production of ROS and RNS and induces DNA damage and the activation of proto-oncogenes (Zhang et al., 2020). It is well-known that HP can accelerate DNA ubiquitination and promote the degradation of p53 by the proteasome (pepsinogen I and II; Wei et al., 2010). In addition, p53 possesses antioncogenic capability; thus, this process exacerbates the intracellular uptake of CagA by T4SS (Wei et al., 2015). Then, a large amount of CagA activates downstream proto-oncogenes. However, it has been observed that patients with gastric cancer exhibit high levels of p53, perhaps because of the stimulation of the body’s immune response in the early stages of the disease. On the other hand, its tumor suppression function is dysregulated in the later stage. Therefore, the regulatory mechanism of p53 in HP causing gastric cancer needs to be further explored.

Cytotoxin-associated gene A may be regulated by VacA, which continuously promotes the carcinogenic process in gastric mucosal cells. The reason for this may be that the cell cycle inhibitor p21 induces senescence in premature cells, a process called “hit and run.” VacA is regarded as double carcinogenesis considering that involves the activation of the ERK and Wnt signaling pathways and the upregulation of proto-oncogene expression along with the occurrence of defects in microtubule mitotic spindle formation through CagA/PAR1b. These changes synergistically increase genomic instability (Molina-Castro et al., 2020) and promote p21 (a cell cycle inhibitor) to counteract the prosenescence process mediated by CagA. Finally, the programmed apoptosis of the cells is disrupted and they tend to be in a hypo-fractionated state. In addition, the MEK-ERK signaling pathway can be used to increase the expression of the antiapoptotic protein, myeloid leukemia sequence-1 (MCL1), thereby providing a pathological basis for the proliferative differentiation of gastric mucosal cells toward abnormal morphology (Su et al., 2016; Lee et al., 2017; Butt et al., 2019).

The TIFA/ALPK1 axis of the bacterial LPS plays a natural immune recognition role (Zhang et al., 2020). When the GHCs are infected by HP, B-ADP-heptose enters the epithelium by means of the HP-T4SS system and binds to ALPK1, which causes the activation of TIFA protein and NF-kb and provides the molecular basis for the release of proinflammatory factors. Nuclear factor red lineage 2-related factor 2 (Nrf2) is an important factor that regulates the encoding of antioxidant enzymes, such as GCLC, GSS, and oxygenase-1 (HO-1; Li et al., 2008), and protects cells from oxidative damage. Under normal conditions, Nrf2 is present in Kelch-like ECH-associated protein 1 (Keap1), which is degraded by the proteasome. Conversely, oxidative stress leads to the separation of Nrf2 from Keap1, making it less susceptible to ubiquitination and translocation to the nucleus (Kang et al., 2004). In the end, Nrf2 binds to the promoter regions (AREs), as a cytolytic agent. If the protease inhibitor PS-341 is used, it protects Nrf2 from being ubiquitinated, thus decreasing the levels of GCLC and GSS. Hence, the effect of the proteasome on Nrf2 is probably responsible for the activation of the signaling pathway of oxidative stress mediated by HP.

It has been reported that oxidative stress also leads to cellular autophagy. The specific mechanism may involve the release of VacA by HP, which in turn induces membrane pore formation that results in energy loss from the mitochondria, and affects the formation of mTORC1 complex, with the onset of autophagy (Figure 1). Furthermore, LRP1 combines with VacA to activate downstream canonical or the non-canonical signaling pathways. Interestingly, LRP1 binds exclusively to the –s1m1 isoform of VacA, but not to its –s1m2 or –s2m2 isoforms (Yahiro et al., 2012). The accumulation of histoproteinase-derived D results in the continuous propagation of HP in the autophagosome (Sit et al., 2020).

## Oxidative Stress Caused by HP Contributes to Gastric Diseases

*Helicobacter pylori* has been defined as a grade-A carcinogen by the International Agency for Research on Cancer (IARC; Anwar et al., 1994), and long-term HP infection is susceptible to the development of malignant intestinal metaplasia in GHCs. HP is not only regarded as a high-risk factor for early gastric cancer but has also been closely associated with the occurrence of peptic ulcer, gastritis, and primary B-cell gastric lymphoma (MALT lymphoma; Camilo et al., 2017). Therefore, HP eradication can effectively reduce the incidence of early gastric cancer (Supajatura et al., 2002; Butcher et al., 2017). In this part, we aim to clarify that the specific molecular mechanisms underlying the release of inflammatory factors and its following oxidative stress in various gastric diseases, such as TGF-β TNF-α, and/or IL-8 and IL-33 (Crowe, 2019; Doulberis et al., 2020).

### Chronic Gastritis

In a study, it was shown that p53 upregulated the modulator of apoptosis (PUMA), as a proapoptotic protein, can mediate acute tissue injury. When the level of PUMA was elevated in the tissues, the apoptosis of GHCs was increased, which induced the aggravation of gastritis and increased the incidence rate of gastric cancer (Dang et al., 2020). However, the outcome of apoptosis in GHCs was reversed in the PUMA knockout mice. Moreover, there was only a little possibility of detecting gastritis in these models. Previous studies have confirmed that PUMA is involved in the TGF-a-mediated apoptosis signaling pathway (Wang et al., 2009). In addition, PUMA activation is mediated by TLR2/NF-kB (Bauer et al., 2006). Latest research has demonstrated that the activation of NF-kB can directly affect p53 to regulate BH3, as a Bcl-2 family member, through an independent mechanism (Chen et al., 2020). Therefore, PUMA is considered to be a critical protein regulating the apoptosis process, which can cause gastritis mediated by the mirRNA-24 signaling pathway (Chen et al., 2020).

### Gastric Ulcer

The specific pathological mechanisms of gastric and duodenal ulcers remain unknown; moreover, gastric ulcer has a higher risk of developing into early cancer when compared with duodenal ulcer (Yamaoka, 2010). The morbidity of duodenal ulcer and gastric ulcer caused by HP infection are 90 and 80%, respectively (Chey and Wong, 2007). However, the detection rate of peptic ulcer in Indonesia is not high, only <20%; conversely, other neighbor countries show different diagnostic rates of peptic ulcer, such as 50.9% in Malaysia and 47.6% in Thailand (Binh et al., 2018). The possible mechanism is that people in different regions have varying DupA subtypes. Early studies have shown that high gastric acid is conducive for the positive expression of DupA and directly affects the release of IL-8 to activate proinflammatory factors, such as NF-kB and AP-1. Besides, high gastric acid can induce the inconsistent morbidity of HP infection. Interestingly, some scholars have confirmed that DupA1 is associated with A2147G clarithromycin (CLR)-resistance for the Iraqi population but not with the secretion of IL-8 by the GHCs (Hussein et al., 2015). Therefore, how DupA1 regulates the downstream signaling pathway needs further investigation.

### Gastric Carcinogenesis

Previous studies have reported that the upregulated expression of p21 in the tissues increases the risk of cancer (Georgakilas et al., 2017). Additionally, p21 is closely related to the poor prognosis of gastrointestinal disease. However, it has been shown that Pim2, as an apoptotic protein, can control the process of oxidative stress *via* ROS. Pim2 also plays a carcinogenic role in the GHCs and results in the poor prognosis of patients. The expression of 12 more biomarkers is associated with the pathological mechanism of gastric cancer, including VCAM-1, β-Catenin, c-Myc, CXCL13, DC-SIGN, EGFR, DAPK1, TIMP3, GRIN2B, SLC5A8, CDH1, and GATA-4. The protein and mRNA levels of the first seven genes are elevated in patients with gastric cancer. Conversely, the levels of the tumor suppressors (e.g., DAPK1, TIMP3, GRIN2B, SLC5A8, and CDH1) are low in the gastric tissues of patients with gastric cancer (George et al., 2020). The above genes participate in triggering the ROS production, and their function is to destroy the balance between antioxidant and oxidant effects, finally leading to the damage of mitochondrial DNA. However, it has been reported that the oxidative stress caused by CagA-positive bacteria is more likely to lead to gastric cancer (Blaser et al., 1995). The possible mechanism is that although CagA induces DNA damage in the GHCs, it does not lead to apoptosis, which greatly aggravates the risk of gastric cancer (Chaturvedi et al., 2011). Furthermore, HP-colonized mucosal cells that have defects in DNA repair mechanisms are more susceptible to oxidative stress and DNA damage (Kidane et al., 2014). In addition, HP colonization destabilizes the regulation of antioxidant proteins, accompanied by epigenetic changes and DNA methylation (e.g., TWIST-1, GATA-4, and GATA-5; Crowe, 2019) as well as micro-RNA dysregulation (e.g., mir-21, mir-27a, mir-92a, mir-146a, mir-155, mir-326, and mir-663; Singh et al., 2019; George et al., 2020). This process mainly involves host cell recognition of the invading pathogens through extracellular toll-like receptors (TLR). Interestingly, MyD88, as a target protein binding to TLR, can be modulated by mirR155, which, in turn, impacts the activation of the downstream signaling cascade (IRAK-1/4 and TRAF6) mediating the NF-kB pathway. At the same time, the mentioned proteins are also affected by miR-146a. Conversely, HP mediates the entry of PGN into the GHCs through the T4SS system and intracellular Nod, which directly activates the NF-kB signaling pathway and causes a proinflammatory immune response (Geddes et al., 2009). miR-27a, which is considered as an oncogenic miRNA in gastric cancer, is known to play a role in inhibiting the protective factors against oxidative stress, such as FOXO1 (Furukawa-Hibi et al., 2002). It has been reported that when the GHCs are infected with HP, the expression level of purine-free/pyrimidine-free nucleic acid endonuclease 1 (APE1) is decreased, and the immune response of the organism is subsequently lowered. The repair ability of the T-cells is significantly weakened, which enhances the probability of DNA carboxy-terminal genomic mutations (Bhattacharyya et al., 2014). APE1, as a DNA repair enzyme, has the ability to regulate epithelial ROS by regulating the interaction of Rac1 and Nox1, which, in turn, inhibits the production of NADPH oxidase, thus reducing the level of ROS (den Hartog et al., 2016). However, when the organism is in the decompensated phase, the protective effect of APE1 will not be able to reverse the DNA damage by ROS. In summary, APE1 can be considered as a target protein for assessing the genomic lifespan; meanwhile, phosphohistone H2AX is a marker of damaged DNA repair. Hence, targeted drug therapy seems to be an effective way to prevent HP-induced base excision or mismatch.

Transforming growth factor-β1 (TGF-β1) is known to be involved in cell proliferation, fibrosis, and other pathological processes. It has been affirmed that the severity of gastritis can be directly proportional to the expression level of TGF-b1. A recent study alluded that TGF-β1 increases the possibility of HP infection and subsequently leads to the activation of the epithelial–mesenchymal transition pathway, which serves as a basis for the development of gastric cancer stem cells (Choi et al., 2015). In addition, activation of the TGF-b signaling pathway induced by ROS directly affects the pathological changes in carcinogenesis. Earlier studies have found that if the genes were mutated in a mouse model, the oxidative stress would occur before the development of the inflammatory response (Touati et al., 2003). It has been indicated that the mutation of the gene triggers the inflammatory response and further exacerbates the gastric ulcers (Sheh et al., 2010). In conclusion, HP is recognized as an oncogenic bacterial pathogen. When patients with advanced gastric cancer have a coexisting long-term HP infection, their 5-year survival rate is low. The underlying mechanism may be that HP can lead to a proinflammatory immune response as well as oxidative stress, both of which synergistically promote gastric tumor formation (Richman et al., 2017). In the future, estimating the HP infection through liquid biopsy might be an important tool for early gastric cancer screening (Tammer et al., 2007).

### Gastric Lymphoma

The majority of gastric MALT lymphomas (92%) are closely associated with HP infection (Gong et al., 2016). The virulence of the HP strains involved in gastric MALT lymphoma is less than that of the strains involved in gastric adenocarcinoma. The reason might be that the latter strains contain the VacA m2 allele without the CagPAI. If CagA is detected, the HP carriers are more likely to progress toward diffuse large B-cell lymphoma (DLBCL; Floch et al., 2017). Moreover, there is a higher rate of detecting t (11;18) mutations (Matsuoka et al., 2020), which usually predicts a poor therapeutic outcome. In the treatment for gastric MALT lymphoma, the addition of antibiotics for efficiently blocking the disease is recommended even if the patient does not carry HP, with a cure rate of 57%. Therefore, prophylactic eradication of HP is very effective in reversing MALT lymphoma, either in the early MALT stage or in the late bone marrow-involvement stage. Nevertheless, it cannot be ignored that MALT lymphoma has the potential to recur; it usually reappears several years after curation, perhaps because the risk factors associated with gastric cancer have not been fundamentally eliminated. Hence, regular endoscopy is advised (Hatakeyama, 2019). Strikingly, nucleotide-binding oligomerization domain protein 2 (NOD2) acts as a pattern-recognition receptor. HP mediates NF-κB signaling activation through NOD2. If the mutation occurs in the R702W gene, the NF-κB signaling pathway is unregulated, which protects the body from the damage of oxidative stress induced by HP (Poplawski et al., 2013). Therefore, it is worthwhile to note the relationship between the NOD2 gene and the pathogenesis of gastric MALT lymphoma (Deng et al., 2017).

## Therapy

Currently, antibiotics, such as metronidazole (MTR), CLR, levofloxacin, and moxifloxacin, combined with gastric mucosal protective agents and acid suppressant quadruple therapy remain the initial choice for the eradication of HP. However, when HP reappeared in GHCs, quadruple therapy was not effective, mainly owing to the presence of antibiotic resistance in HP (De Francesco et al., 2010). A previous study confirmed that the long-term use of proton pump inhibitors (PPIs) increases the risk of gastric cancer in patients with or without HP eradication (Seo et al., 2021). Considering the pathogenesis of drug resistance influenced by multiple factors, new targeted antimicrobial therapies are deemed more suitable for individualized treatment that could effectively avoid drug resistance in clinical practice as well as decrease the phage for PPI use. Therapeutic drugs for HP infection can be divided into various categories. One category of drugs controls the damage to the gastric mucosa caused by HP by targeting the inhibition of virulence factors. Another drug-therapy focus on the inhibition of the whole process of bacterial infection, such as probiotics combined with antibiotic therapy and bacteriophage therapy. Furthermore, since the persistent infection and colonization of HP depends on the genotype and the individual’s polymorphisms of the different strains, it might be more effective to explore individualized treatments for specific populations. Thus, antioxidant therapy plays a significant role in controlling HP infection, involving targeted inhibition of oxidative stress-mediated signaling pathways. The above evidence suggests that antioxidant therapy may serve a landmark advancement for HP eradication when compared with the traditional eradication methods. Notably, antioxidant therapy is the focus of this article, which involves targeted inhibition of the signaling pathways during HP-induced oxidative stress.

### Novel-Targeted Antibiotic Therapy

Nammi et al. (2017) screened and analyzed 23 HP strains using a five-step *in silico* analysis. Moreover, the results revealed that 31 pathogenic islands were associated with the signaling domain editing the gene encoding the virulence. For instance, VacA plays an important pathogenic role in gastric diseases, which is contributed to long-term infection of the HP strain. This process can not only prevent from the self-defense effect of the host cell, but also resist the action of antibiotics, achieving drug evasion. VacA, which acts as a co-stimulator of CagA-phosphorylated T cells, mediates the activation of the downstream pathogenic mechanisms (Ansari and Yamaoka, 2020). Thus, the inhibition of this gene could play a role in reducing the virulence effect of pathogenic islands and act as a potential drug target for new antibiotic therapies, instead of the conventional antimicrobial modalities (Nammi et al., 2017). Past studies have demonstrated that HP exerts an unusual cluster of OMPs, signifying its ability to adapt to unique gastric environment (Alm et al., 2000). Perhaps the interference with the expression of OMPs could serve as a target antigen for DNA multivalent vaccine construction (Vale and Oleastro, 2014). In addition, these novel therapies are focused on the inhibition of signaling pathways involved in the biological process, such as cell-wall integrity and synthesis, replication of nucleic acids, and mitochondrial metabolism. For example, coenzyme (CoA) is a cofactor of bacterial colonization that supports the synthesis of phosphopantetheine adenylyltransferase (PPAT). The targeted inhibition of PPAT can efficiently block the activity of HP. Furthermore, the succinylase pathway is the only lysine synthesis pathway, and lysine serves as an essential element in the cell wall of bacteria. When antibiotics directly inhibit the key proteins in the process of lysine synthesis, such as N-succinyl-L, L-diaminoacrylic acid desuccinate lyase (SDAP deacylase; DapE), they would be able to disrupt the cell wall of HP (Karita et al., 1997; Mandal and Das, 2015).

### Probiotic Therapy

The combination of probiotics and antibiotics can target the floating bacteria and also block the recurrent infection caused by recalcitrant bacteria, achieving a dual benefit in the treatment (Espinoza et al., 2018; Ji and Yang, 2020). According to a meta-analysis, most probiotics are considered to represent the colonization of the human digestive tract, such as *Lactobacillus* and *Bifidobacterium* (Wen et al., 2020). It can fight HP either directly or indirectly *via* the following mechanisms: organic acids, short-chain fatty acids, and antimicrobial peptides secreted by probiotics can directly inhibit HP infection. Moreover, secreted IgA is produced to improve the defense function of gastric mucosa. Furthermore, the barrier function is systematically enhanced by probiotics, which improves immunity against HP infection (Dargenio et al., 2021). It is well-known that a probiotic and prebiotic-rich diet is one of the main combination therapies for the modulation of intestinal health (Cruz et al., 2021). It has been known to efficiently inhibit the process of oxidative stress and inflammation, following the increased level of claudin-3 and occludin, which contributes to the restoration of the gastrointestinal barrier function (Cruz et al., 2020). Therefore, the optimal combination therapy not only reduces the adverse effects and drug resistance caused by antibiotic overdose but also maximizes the effect of killing HP, which has a significantly higher HP eradication rate (84.1 vs. 70.5%), with a significantly lower incidence of adverse events (14.4 vs. 30.1%) when compared with the normal antibiotic therapy (Huang et al., 2015). Accordingly, we hypothesized that the two-combination therapy is excellent in eradicating HP (Seyler et al., 2001). Another report concluded that the addition of plant extracts to a mixture of probiotics and antibiotics, as an emerging therapy, was effective in improving the killing of HP (Chey et al., 2017). However, it remains unknown whether its sterilizing effect could be superior to the mixture of probiotics and antibiotics without the addition of plant extracts.

### Phages Therapy

Prephages, present in the strains of HP, possess a recombinant double gene with a high diversity, which considerably contributes to the persistence of HP colonization in different populations. It has been demonstrated that phages can be easily isolated with high specificity, which has renewed interest in the alternative therapy. Moreover, phage therapy has confirmed to be effective against a wide range of bacterial infections as well as a chronic course of infection. When compared with the conventional antibiotic therapy, phages have demonstrated obvious advantages in controlling HP colonization without affecting the other normal flora, which may be explained by the fact that phages only transcriptionally replicate at the site of HP infection and without other side-effects. Another option that has been presented is to utilize phage-cleavage proteins, such as lysine, which play the role of lysing the cell wall of host bacteria. However, the modified lysine limits the antimicrobial capacity toward overcoming the limitations of the Gram-negative outer membrane (Lukacik et al., 2012). There are only a few studies on the application of phages in the field of HP eradication, and the specific effect remains unknown in clinic settings.

### Antioxidant Therapy

It has been reported that GSH levels and GSSG/GSH ratio are significantly lower in patients with gastric cancer if they are coinfected with HP. The levels of glutamine are also low. Furthermore, hydrogen peroxide production is promoted, and oxidative stress is aggravated. However, the addition of GSH treatment was effective in alleviating the excessive accumulation of ROS (Lee et al., 2015). In experiments with AGS cells infected with HP strains, the GSH levels were lower in patients with gastric cancer than in those with gastric or duodenal ulcer, suggesting a more severe oxidative stress response resulting from the development of HP into gastric cancer (Chitcholtan et al., 2008). A recent study found that a component called S-allyl cysteine, extracted from garlic, plays an anti-inflammatory and antioxidant role. The study showed that this compound significantly increased the GSH level in the stomach, liver tissue, and serum of gastric cancer rat models and alleviated the risk of gastric cancer in the organism (Velmurugan et al., 2003). In conclusion, low levels of GSH induced intestinalization in the GHCs. Hence, increasing the GSH level may protect the rats from the risk of HP-induced gastric mucosal carcinogenesis and block oxidative stress damage (Matsuoka et al., 2020).

It has been stated (Toh and Wilson, 2020) that vitamin C deficiency is also a trigger that contributes to oxidative stress. The possible mechanism could be explained by the fact that vitamin C blocks the activation of NF-κB/STAT3 by HP and inhibits the proliferation and differentiation of the AGS cells by upregulating epidermal growth factor (EGF)-like transmembrane proteins and two follicle inhibitory motifs 2 (TMEFF2). Moreover, the mitochondria-mediated AGS apoptotic pathway plays a role in suppressing intestinalization of the GHCs (Han et al., 2019). On the other hand, *in vitro* studies have shown that β-carotene decreases the oxidative stress by suppressing NADPH oxidase, resulting in low ROS levels, which, in turn, interrupts the activation of NF-kB and protects the TNF receptor-related factor (TRAF, TRAF1, and TRAF2) genotypes from nuclear translocation and nuclear transcriptional targets. This process prevents the proliferation and differentiation of AGS cells induced by TRAF genes. In clinical trials, it has been demonstrated that regular oral administration of β-carotene is beneficial in reducing the bacterial colonization by 48% (Park et al., 2019). Recently, it has been identified that α-lipoic acid (α-LA) is a naturally occurring dithiol with antioxidant and anti-inflammatory functions. This process was mainly focused on decreasing the interaction between Nrf2 and Keap1, which inhibits the production of the proinflammatory cytokine IL-8 and reduces the infection of the AGS cells by the Nrf2/HO-1 pathway (Kyung et al., 2019). Accordingly, it has been suggested that the dietary intake of foods rich in vitamin C, carotenoids, and α-LA may reduce the morbidity of gastric disease associated with HP infection (Kyung et al., 2019). Clinical guidelines recommend the inclusion of the abovementioned additives in the diet on a daily basis, with the prospect of completely curing HP infection. Omega-3 fatty acids can increase the anti-inflammatory and antioxidant properties of antioxidants and prevent the polyunsaturated long-chain fatty acids from being oxidized (Sepidarkish et al., 2019).

However, omega-3 may cause oxidative stress in the body, and the mechanism is associated with the decreased expression of antioxidant enzymes. Hence, it is recommended that an ideal therapy needs to be adopted, which includes antibiotics for neutralizing the oxidative effect of omega-3, including CLR, MTR, quinolones, amoxicillin (AMX), and tetracycline.

Last but not the least, there is a need to block the entire process of oxidative stress. For example, the *in vivo* regulator of homeostatic stress, HsrA, is a unique regulator for epsilon proteobacteria, participates in modulating the protein expression and redox homeostasis. Thus, it is considered to be a promising therapeutic target for eliminating HP (Pelliciari et al., 2017; González et al., 2019a,b). Additionally, the combination of curcumin and Res can effectively control the high expression of apoptosis-regulated genes such as PMAIP1, BID, ZMAT3, and FAS in the GHCs of patients with HP infection, leading to a reduction in the apoptosis levels (Xu et al., 2018; Gavrilas et al., 2019).

## Conclusion

*Helicobacter pylori* infection triggers a prolonged inflammatory response in the gastrointestinal mucosa through its virulence factors. However, this response activates the immune system to destroy the bacteria, followed by a chronic oxidative stress process. In fact, this process has little effect on the elimination of strains and provides a microenvironment for HP to colonize the surface of the GHCs in the long run. Subsequently, the process of chronic oxidative stress damages the immune system, such as the mismatch of the DNA chain and the epigenetic abnormalities that induce the proliferation and differentiation of the GHCs, finally causing moderate and severe intestinal metaplasia (Singh et al., 2019). To effectively manage the digestive diseases triggered by HP strains, it is essential to identify the specific pathological mechanism mediated by HP. This review has outlined the molecular mechanism of oxidative stress induced by HP as well as the typical biomarkers, and has further identified the critical proteins involved in the regulation of the signaling pathways. According to the findings, targeted therapy against oxidative stress administered in combination with antibiotics may serve as a promising strategy for preventing or delaying the gastric mucosal diseases caused by HP infection in the future (Singh et al., 2019).

## Author Contributions

LH and JW proposed and collected the structure and quality of the manuscript, compared the published data, and abstracted the paper. LH summarized the review research from PubMed and wrote the first manuscript draft. All authors contributed to the article and approved the submitted version.

## Funding

This work was supported by the scientific research and cultivation of young talents in The First Affiliated Hospital of Nanchang University (No: PRJ-20211002213746346).

## Conflict of Interest

The authors declare that the research was conducted in the absence of any commercial or financial relationships that could be construed as a potential conflict of interest.

## Publisher’s Note

All claims expressed in this article are solely those of the authors and do not necessarily represent those of their affiliated organizations, or those of the publisher, the editors and the reviewers. Any product that may be evaluated in this article, or claim that may be made by its manufacturer, is not guaranteed or endorsed by the publisher.
